# Whole-Blood Flow-Cytometric Analysis of Antigen-Specific CD4 T-Cell Cytokine Profiles Distinguishes Active Tuberculosis from Non-Active States

**DOI:** 10.1371/journal.pone.0017813

**Published:** 2011-03-15

**Authors:** Urban Sester, Mathias Fousse, Jan Dirks, Ulrich Mack, Antje Prasse, Mahavir Singh, Ajit Lalvani, Martina Sester

**Affiliations:** 1 Department of Internal Medicine IV, Saarland University, Homburg, Germany; 2 Department of Transplant and Infection Immunology, Institute of Virology, Saarland University, Homburg, Germany; 3 Department of Internal Medicine V, Saarland University, Homburg, Germany; 4 Department of Pneumology, University Medical Center Freiburg, Freiburg, Germany; 5 Helmholtz Center for Infection Research, and Lionex Diagnostics & Therapeutics GmbH, Braunschweig, Germany; 6 Tuberculosis Research Unit, Department of Respiratory Medicine, National Heart and Lung Institute, Imperial College London, London, United Kingdom; Statens Serum Institute, Denmark

## Abstract

T-cell based IFN-γ release assays do not permit distinction of active tuberculosis (TB) from successfully treated disease or latent *M. tuberculosis* infection. We postulated that IFN-γ and IL-2 cytokine profiles of antigen-specific T cells measured by flow-cytometry *ex vivo* might correlate with TB disease activity *in vivo*. Tuberculin (PPD), ESAT-6 and CFP-10 were used as stimuli to determine antigen-specific cytokine profiles in CD4 T cells from 24 patients with active TB and 28 patients with successfully treated TB using flow-cytometry. Moreover, 25 individuals with immunity consistent with latent *M. tuberculosis* infection and BCG-vaccination, respectively, were recruited. Although the frequency of cytokine secreting PPD reactive CD4 T cells was higher in patients with active TB compared to patients with treated TB (median 0.81% vs. 0.39% of CD4 T cells, p = 0.02), the overlap in frequencies precluded distinction between the groups on an individual basis. When assessing cytokine profiles, PPD specific CD4 T cells secreting both IFN-γ and IL-2 predominated in treated TB, latent infection and BCG-vaccination, whilst in active TB the cytokine profile was shifted towards cells secreting IFN-γ only (p<0.0001). Cytokine profiles of ESAT-6 or CFP-10 reactive CD4 T cells did not differ between the groups. Receiver operator characteristics (ROC) analysis revealed that frequencies of PPD specific IFN-γ/IL-2 dual-positive T cells below 56% were an accurate marker for active TB (specificity 100%, sensitivity 70%) enabling effective discrimination from non-active states. In conclusion, a frequency lower than 56% IFN-γ/IL-2 dual positive PPD-specific circulating CD4 T-cells is strongly indicative of active TB.

## Introduction

Interferon-γ release assays (IGRA) are transforming diagnosis of latent tuberculosis infection and improving diagnostic evaluation of patients with active tuberculosis (TB) [1]. IGRA use IFN-γ as the sole read-out and provide only limited biological information which is clinically interpreted in a binary fashion, whereby a result indicates either presence or absence of infection. Moreover, although IGRA responses are quantitatively higher in untreated active TB than in treated TB [2], [3], the considerable overlap in responses between these patient groups precludes use of IGRA to distinguish between these distinct states. According to a recent meta-analysis, the sensitivity of IGRAs for diagnosis of active TB is higher than tuberculin skin-testing, but the specificity of IGRAs to distinguish between patients with active TB and non-active TB suspects is still inadequately low [4].

The cellular immune response to intracellular pathogens comprises a spectrum of T-cell subpopulations characterized by distinct cytokine secretion profiles and cell surface marker phenotypes. Three main subsets are recognized and can be identified on the basis of T-cell cytokine profiles: effector T cells that secrete only IFN-γ, effector-memory T cells that secrete both IFN-γ and IL-2 and central-memory T cells that secrete only IL-2. The relative proportions of these three T cell subsets correlate with antigen load in chronic viral infections [5], [6]. Millington et al have previously found that the relative proportions and frequencies of these three T-cell subsets among *M. tuberculosis* antigen-specific T cells correlated with pathogen burden and antigen load in TB patients. The significant shift in cytokine profiles after anti-TB treatment led the authors to propose this as a new approach for monitoring anti-mycobacterial treatment but the technical approach was labour-intensive and the number of patients was small [7].

We used a rapid 6 h-whole blood assay for intracellular cytokine staining of IFN-γ and IL-2 and flow-cytometry to measure and compare antigen-stimulated T-cell cytokine profiles in 24 patients with active untreated TB and 28 patients who had been successfully treated. A threshold in IFN-γ/IL-2 dual positive CD4 T cells was established to distinguish active disease from successfully treated disease. This threshold was further assessed in 50 healthy individuals without evidence for active tuberculosis. Among those, 25 had a antigen-response profile compatible with latent *M. tuberculosis* infection and 25 individuals had an immune response consistent with BCG vaccination or infection with non-tuberculous mycobacteria (NTM).

## Materials and Methods

### Subjects

Twenty-four patients with active tuberculosis and 28 patients with successfully treated tuberculosis were prospectively recruited at the university hospitals of the Saarland and of Freiburg, Germany (table 1). All patients diagnosed with active tuberculosis had positive cultures for *M. tuberculosis* or acid fast bacilli from one or more clinical specimens, or clinical and radiologic features highly suggestive of tuberculosis together with a good response to anti-tuberculosis treatment (table 1). None had started treatment at the time of enrolment. Patients with successfully treated tuberculosis were studied 35.5±20.4 years (interquartile range 20–50 years) after treatment. Importantly, those patients had not suffered from relapse and did not show any signs or symptoms compatible with active tuberculosis at the time of analysis. Moreover, 25 healthy individuals (40.3±11.6 years of age, 10 males, 15 females) with an immune status compatible with latent infection with *M. tuberculosis* were recruited. This classification was based immunologically on the presence of specific immunity towards PPD and at least one of the *M. tuberculosis* specific antigens ESAT-6 and CFP-10 [8]. In addition, 25 healthy individuals with a positive immune response towards PPD in the absence of specific immunity towards ESAT-6 and CFP-10 were analysed (43.3±12.7 years of age, 9 males, 16 females). Although the BCG-vaccination status was not consistently known and this does not firmly exclude latent infection, this very commonly observed response pattern would be consistent with either BCG-vaccination or prior NTM infection. These 50 individuals did not have any known contact to a patient with active tuberculosis and did not show any signs or symptoms of active disease. Medical history for HIV infection was negative in all tested individuals; specific testing, however, was only performed if clinically indicated.

**Table 1:** Demographic and clinical characteristics of patients with active and successfully treated tuberculosis.

	Patients with	
	Active TB	Successfully treated TB
	n = 24	n = 28
**Demographic characteristics**		
Mean age (years)	46.7±18.7	58.7±14.2
Sex (male/female)	9/15	21/7
Ethnicity		
– white	19	28
– asian	4	-
– indian	1	-
**Clinical characteristics of patients with disease**		
Location of disease		
– Pulmonary	19*	
– Extrapulmonary, among those	5	
Lymphatic	1	
Organ/bone	1	
Skin	1	
Disseminated	2	
**Basis of diagnosis**		
– Culture positive for MTB	12	
– Acid fast bacilli on microscopy	9	
– Response to treatment	3	
Mean time after treatment (years)	0, treatment naïve	35.5±20.4
**Immune-based tests**		
PPD positive; number (percentage)	23 (95.8%)	23 (82.1%)
ESAT-6 and/or CFP-10 positive; number (percentage)	16 (66.7%)	16 (57.1%)

*including one patient with pleural tuberculosis.

### Ethics statement

The study was approved by the local ethics committee (Ärztekammer des Saarlandes), and written informed consent was obtained from all participants.

### Quantitation and characterization of antigen-specific CD4 T cells within whole blood

Specific stimulation of CD4 T cells was performed directly from heparinized whole blood for a total of 6 h. Titered amounts of PPD (7.32 µg/ml, Tuberkulin for in vitro use (RT-50); Statens Serum Institute, Copenhagen, Denmark), recombinant ESAT-6, and recombinant CFP-10 (10 µg/ml each, Lionex, Braunschweig, Germany) were used as stimuli as previously described [9]–[12]. Each antigen was stimulated separately and a total volume of 300 µl was used for each stimulus. As negative control, cells were stimulated with diluent (phosphate buffered saline). Stimulation of all samples including negative controls was done in the presence of 1 µg/ml anti-CD28 and anti-CD49d (clones L293 and 9F10; BD, Heidelberg, Germany), respectively as described before [5], [9]–[11]. In line with previous observations with other antigens [13], the addition of these antibodies to stimulatory reactions with PPD led to a 1.61±0.40 fold increase in the percentage of IFN-γ positive CD4 T cells and a 1.58±0.39 fold increase in IL-2 positive CD4 T cells (n = 7 individuals, data not shown). Multiparameter staining was performed using anti-CD4 (clone SK3), anti-IFN-γ (clone 4S.B3), anti-IL-2 (clone MQ1–17H12), and anti-CD69 (clone L78; all antibodies from BD). At least 15.000 CD4 T cells were analyzed on a FACS Calibur (BD) using Cellquest Pro 4.0.2 software. T cells responding with cytokine production were CD4 positive. Although CD8 T cells were not stained directly, there was no detectable cytokine production among CD4 negative lymphocytes, indicating that CD8 T cells do not seem to contribute to cytokine production in this experimental setting. Although reactivity in the control stimulations was largely negligible, the percentage of specific T cells was calculated by subtracting the frequency obtained by the control stimulation. The lower limit of detection was 0.05% as previously established [14].

### Statistical analysis

Statistical analyses were performed using Prism V5.03 software (Graphpad, San Diego, USA). Receiver operator characteristics (ROC) analyses were performed to establish cutoffs of PPD reactive CD4 T-cell frequencies and IFN-γ/IL-2 dual cytokine producing CD4 T cells. The cutoff with the highest diagnostic sensitivity and specificity was determined using Youde index statistics. The non-parametric Mann-Whitney test was used to compare PPD reactive T-cell frequencies as well as the percentage of dual cytokine secreting cells between patients with active disease and individuals with successfully treated tuberculosis. If parameters were compared between more than two groups, the non-parametric Kruskall-Wallis test was used.

## Results

**Figure 1:**
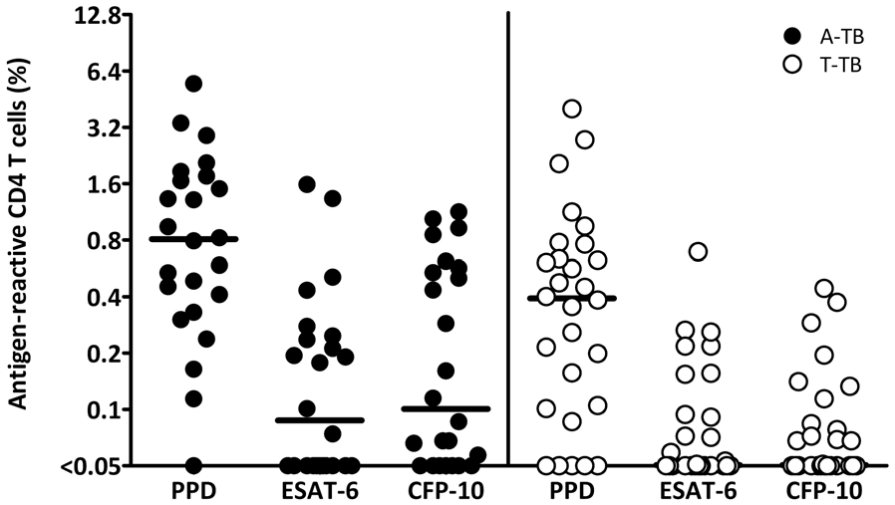
The frequency of antigen-reactive CD4 T cells does not allow distinction between patients with active tuberculosis (A-TB) and successfully treated disease (T-TB). Whole blood from all individuals was stimulated with PPD, ESAT-6, or CFP-10 and antigen-specific CD4 T cells co-expressing CD69, IFN-γ and/or IL-2 were quantified using flow-cytometry. Although the difference in PPD reactive CD4 T-cell frequencies between the two groups was significant (p = 0.02, Mann-Whitney test), it was impossible to correctly assign the patients' clinical status on an individual basis. The median is depicted by the line.

To analyse immune response parameters that allow distinction between patients with active tuberculosis and non-active states, patients with active tuberculosis were first compared with a group of individuals with a history of active tuberculosis where a prior infection was definitely proven. Patients were included several years after successful treatment. Together with the fact that none of them had a tuberculosis relapse, this indicates that the likelihood of overt remaining bacterial activity was rather low. Demographic and clinical characteristics of the 24 patients with active tuberculosis and 28 patients with successfully treated tuberculosis are summarized in table 1. Whole blood from all individuals was stimulated with PPD, ESAT-6 and CFP-10, and antigen-specific CD4 T cells co-expressing CD69, IFN-γ and/or IL-2 were quantified using flow-cytometry. Among patients with active disease, all except one (who had been on corticosteroids for 19 months) showed detectable T-cell reactivity towards PPD (95.8%), whereas only 16 out of 24 individuals (66.7%) were reactive towards the *M. tuberculosis* specific antigens ESAT-6 or CFP-10. Among the 28 individuals with successfully treated tuberculosis, 23 (82.1%) reacted towards PPD, and only 16 (57.1%) were positive towards ESAT-6 or CFP-10 (table 1 and figure 1). In general, specific T-cell frequencies towards ESAT-6 and CFP-10 were lower as compared to those towards PPD. Interestingly, patients with active tuberculosis had higher frequencies of PPD reactive cytokine-positive CD4 T cells (median 0.81%, interquartile range (IQR) from 0.35–1.73%) than individuals with successfully treated disease (median 0.39%, IQR 0.10–0.73%, p = 0.02, Mann-Whitney test, figure 1). However, as the overlap in T-cell frequencies between the groups was substantial, it was impossible to correctly assign the patients' clinical status on an individual basis (area under the curve AUC = 0.63, 95% confidence interval (CI) 0.46 to 0.79). At the optimum cutoff of >0.79% PPD reactive CD4 T cells, the sensitivity and specificity for untreated active TB were as low as 78% (95% CI: 56–93%) and 52% (95% CI: 31–73%), respectively. Thus, neither the frequency of cytokine-positive PPD reactive CD4 T cells nor reactivity towards ESAT-6 or CFP-10 allowed for a distinction between patients with active tuberculosis from successfully treated patients.

**Figure 2:**
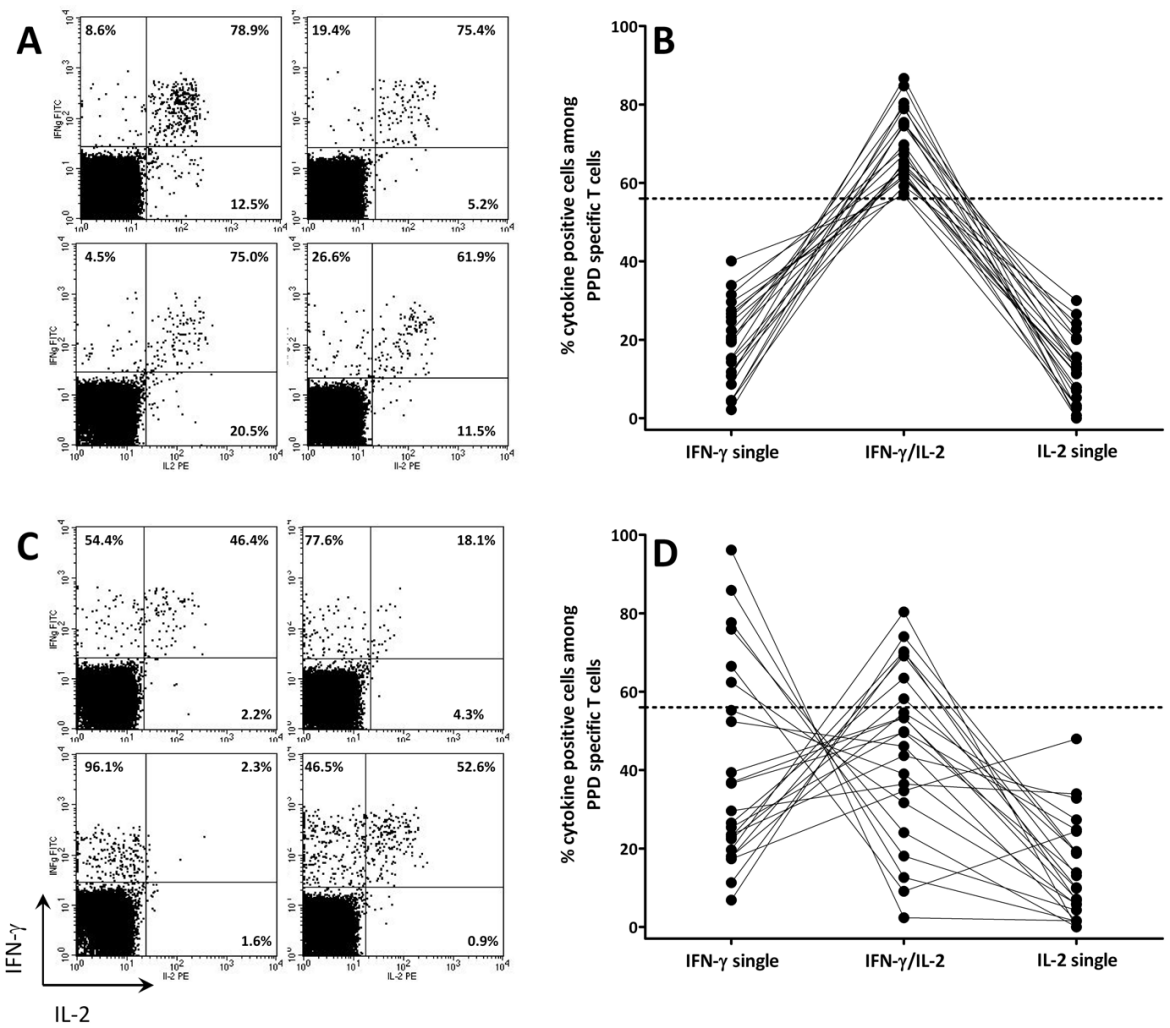
Altered functional signature in patients with active tuberculosis. Typical examples of cytokine profiles of PPD reactive CD4 T cells in patients with (**A**) successfully treated tuberculosis and (**C**) patients with active tuberculosis. Stimulated CD4 T cells were stratified into cells expressing IFN-γ only (upper left quadrant), IL-2 only (lower right quadrant), and dual-cytokine producing cells (upper right quadrant). The numbers in the three quadrants refer to the relative percentage of each cell population. These percentages are displayed for all patients with successfully treated tuberculosis (**B**) and active tuberculosis (**D**). The stippled line indicates the 56% threshold established by ROC analysis.

In an attempt to improve diagnostic specificity for active TB, PPD reactive CD4 T cells were stratified according to their cytokine profile into populations secreting IFN-γ only, IL-2 only and dual-cytokine-secreting cells (figure 2). Stratification into these subpopulations was only feasible in individuals who had PPD reactive CD4 T-cell frequencies above the detection limit (23 of 24 patients with active tuberculosis and 23 of 28 patients with successfully treated tuberculosis). Among successfully treated patients, the cytokine profile was homogeneous and predominantly consisted of IFN-γ/IL-2 double-positive cells (figure 2A and B). In contrast, the percentage of dual cytokine-secreting cells in patients with active disease was significantly lower (p<0.0001, Mann-Whitney test) and the profile showed a strong shift towards cells expressing IFN-γ only (figure 2C and D), whereas the percentage of IL-2 only cells was similar between the groups (figure 2B and D). A receiver operator characteristics (ROC) analysis revealed that a percentage of less than 56% of dual cytokine-secreting cells identified patients with active tuberculosis with a specificity of 100% (95% CI: 85–100%) and a sensitivity of 70% (95% CI: 47–87%; AUC 0.84, 95% CI: 0.72–0.96).

**Figure 3:**
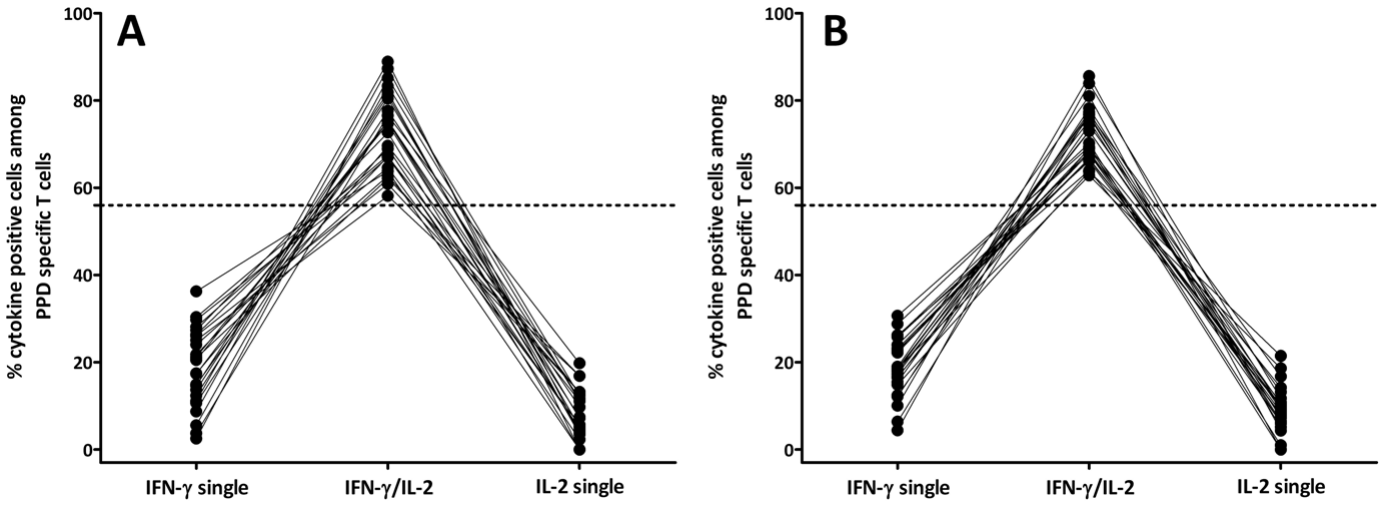
Predominance of dual positive cytokine producing cells in non-active disease states. The percentages of cells expressing IFN-γ only, IL-2 only, and dual-cytokine producing CD4 T cells after PPD specific stimulation in individuals with immunity consistent with latent *M. tuberculosis* infection (**A**) or BCG vaccination/NTM infection (**B**) were determined as described in figure 2.

In a clinical setting, other individuals with non-active states such as subjects with latent *M. tuberculosis* infection or individuals after BCG vaccination or NTM infection may also typically present with immunity towards PPD and/or the *M. tuberculosis* specific antigens ESAT-6 and CFP-10. Based on the results shown in figure 2, we hypothesized that these individuals would show a similar cytokine profile as patients with successfully treated tuberculosis. To assess this hypothesis, the cytokine profile of PPD reactive CD4 T cells among 25 healthy individuals showing concomitant reactivity towards ESAT-6 and/or CFP-10 (consistent with latent *M. tuberculosis* infection) and 25 subjects without ESAT-6/CFP-10 specific immunity (consistent with BCG vaccination or NTM infection) was analysed. The median frequencies of cytokine-positive PPD reactive CD4 T cells in latently infected individuals was 0.48% (range from 0.14–2.57%) whereas it was lower in ESAT-6/CFP-10 negative individuals (0.19%, range from 0.11–1.55%, p = 0.002). When assessing the cytokine profile, the percentage of dual-positive CD4 T cells was above the threshold of 56% in all cases with latent *M. tuberculosis* infection (figure 3A) and with BCG vaccination response/NTM pattern (figure 3B). When ROC analysis performed with patients with active disease as compared to all individuals with non-active states (n = 73), a percentage of less than 56% dual cytokine-positive CD4 T cells again was the optimum cut-off that revealed a sensitivity of 70% (95% CI 47–87%) and a specificity of 100% (95% CI 95–100%; AUC 0.87, 95% CI: 0.77–0.97).

**Figure 4:**
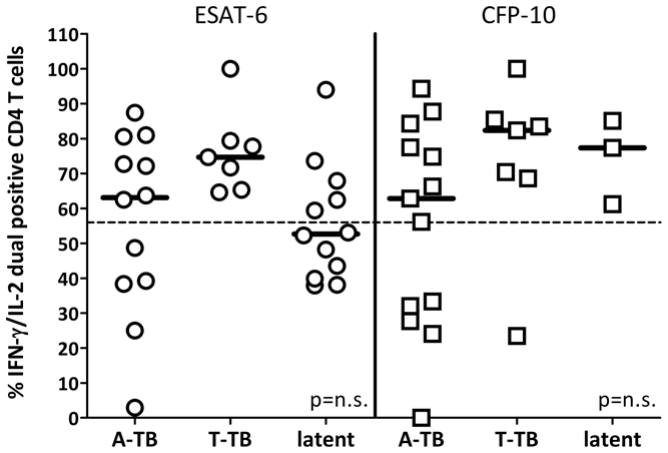
The percentage of ESAT-6 and CFP-10 specific IFN-γ/IL-2 dual positive CD4 T cells does not allow clear distinction between active tuberculosis (A-TB) and non-active disease states (T-TB or latent *M. tuberculosis* infection). This is evident from the fact that 7 of 12 (58%) of latently infected subjects had <56% IFN-γ/IL-2 dual positive ESAT-6-specific CD4 T cells The percentage of dual positive CD4 T cells specific for ESAT-6 and CFP-10 was determined as described in figure 2. As compared with PPD specific profiles with higher overall frequencies, robust determination of profiles specific for ESAT-6 and CFP-10 could only be determined if overall frequencies of specific cells were above 0.1% (i.e. in 62.5% of patients with active tuberculosis, 35.7% of individuals with successfully treated disease and 56.0% of latently infected individuals). The stippled line indicates the 56% threshold established for PPD specific T cells.

Of note, due to a generally lower frequency of ESAT-6 and CFP-10 reactive T cells (see figure 1), robust cytokine profiles in response to the *M. tuberculosis* specific antigens could only be determined in 62.5% of patients with active tuberculosis, 35.7% of individuals with successfully treated disease and 56.0% of latently infected individuals. Interestingly, unlike PPD profiles, there was no significant difference in the percentage of IFN-γ/IL-2 dual cytokine positive CD4 T cells in response to ESAT-6 or CFP-10 among the three groups of individuals and discrimination between active and non-active disease states was poor (figure 4).

Taken together, detection of less than 56% PPD specific dual cytokine-secreting T-cells is strongly indicative of active tuberculosis, whereas frequencies of PPD reactive dual cytokine-secreting T-cells above 56% were observed in all non-active disease states as well as active TB patients.

## Discussion

We found that the cytokine profiles of PPD specific T cells differed significantly between untreated patients with active TB disease and non-active states. Non-active subjects had significantly lower frequencies and a narrower range of PPD specific IFN-γ only-secreting T cells and significantly higher frequencies of dual IFN-γ and IL-2-secreting T cells. In contrast, the frequencies and range of IL-2 only-secreting T cells were similar in the untreated and non-active disease groups. The fact that all successfully treated patients and all other individuals with non-active states had frequencies of dual IFN-γ and IL-2-secreting PPD specific T cells above 56% means that frequencies of these T cells below 56% were only seen in untreated patients, making this a 100% specific marker for active disease. Frequencies of dual cytokine-secreting T cells of less than 56% were only 70% sensitive for active tuberculosis because 7 untreated patients had frequencies of these T cells above 56%. There were no clinical, radiological or microbiological features that clearly distinguished these 7 patients from the remaining 16 who had frequencies of dual cytokine-secreting T cells below 56%, although it is notable that 4 of the 7 had paucibacillary disease manifesting as cutaneous TB, bone TB, pleural TB and lymph node TB. The remaining three had bilateral sputum culture-positive pulmonary TB, consistent with higher bacterial loads.

Relapse or re-infection in patients with previously treated TB is not uncommon and is clinically very difficult to distinguish from alternative pyrexial or respiratory illnesses when they present in previously treated TB patients [15]. Our findings could potentially help to determine whether or not previously treated patients have active TB at the time of clinical presentation and diagnostic work-up. However, before clinical application, our results including the 56% cutoff of dual-positive T cells need first to be confirmed in larger numbers of patients. This would also have to include TB suspects with a lung disease different from tuberculosis or patients in the setting of contact tracing. Moreover, the kinetics of the shifts in cytokine profile under treatment need investigation as all our cured patients had been treated many years or decades previously. We therefore could not determine how soon after treatment the shifts in cytokine profile take place although Millington et al noted significant differences already in patients 3–6 months after treatment initiation [7], suggesting the potential of this approach for treatment monitoring. However, their study and ours were cross-sectional and determining the clinical utility of cytokine profiles for treatment monitoring will require a larger, longitudinal study.

We have chosen individuals with a proven history of tuberculosis several years after treatment to decrease the likelihood of overt remaining bacterial activity. Given that successfully treated and latent TB are characterized by a lower antigen load than active disease, we postulated that the cytokine profile of latent TB infection was similar to that of our treated TB group. Our results confirmed this hypothesis and suggest that cytokine profiling could make it possible immunologically to distinguish not only between active and successfully treated TB, but also between active disease and latent *M. tuberculosis* infection or vaccination responses. In our analyses we have applied currently used immune-based estimates to stratify potentially latently infected subjects and potentially vaccinated subjects (see figure 3A and B); however, this distinction is less important when exploring an immune-based test to discriminate active disease from any non-active state, as the cytokine profiles from all non-active states were different from those observed in active tuberculosis. In support of this, two recent studies indicate that a preserved capacity to produce IL-2 after overnight or 72 h of stimulation is a typical feature of latent *M. tuberculosis* infection [16], [17]. The non-active control subjects analysed in our study were tested in the absence of any known recent tuberculosis exposure. Based on our findings, it will be of considerable interest to determine whether the cytokine profile can be altered in suspects of active tuberculosis or in a setting of contact tracing; if so, this could indicate a state of increasing bacterial burden and consequently higher risk to develop active tuberculosis and could represent a promising method that should be explored to target tuberculosis preventive chemotherapy.

The whole blood approach has the potential to differentiate active from treated or latent TB on an individual patient basis within one working day. The assay simultaneously measures IFN-γ and IL-2 at the single cell level which is neither possible by IFN-γ ELISpot nor by ELISA, the two platforms used in the current commercially available IGRAs. However, measurement of several soluble cytokines by multiplex cytometric bead array in antigen-stimulated supernatants or unstimulated plasma has identified combinations of cytokines, and individual cytokines such as IP-10 and IL-6, that differ significantly between untreated and treated patients but with considerable overlap in responses between the groups [18]. Two recent studies have assessed IFN-γ, IL-2 and TNF-α induction after overnight stimulation of whole blood or PBMC from patients with active tuberculosis and latently infected individuals [19], [20]. Although both studies focused on multifunctionality of specific T cells to distinguish active disease from latent infection, the sensitivity and specificity of this approach was not explicitly assessed. Interestingly, however, in line with our data and a recent study by Casey *et al.* [21], their results presented indicate that the percentage of IFN-γ/IL-2 double positive T cells was higher in latently infected individuals; in that respect, it would be interesting to comparatively assess sensitivities and specificities for multifunctional and for IFN-γ/IL-2 double positive T cells to distinguish active from latent infection. Apart from cytokine profiling, other immune-based approaches exist to distinguish active disease from non-active states, such as the comparative analyses of *M. tuberculosis* specific T-cell responses from blood and specimens from the sites of disease. However, these approaches are invasive and depend on the availability of specimens from the sites of disease [22]–[26].

Changes in cytokine profiles could be a general feature of *M. tuberculosis* specific T cells in active disease. In this study, however, cytokine profiles of ESAT-6 and CFP-10 specific T-cells did not significantly differ between untreated and treated patients, in contrast to other recent studies. The reasons for this are unclear but the frequencies of ESAT-6 specific and CFP-10 specific T cells in the present study were determined *ex vivo* without enrichment and were often near the threshold for detection. Other studies may have detected lower frequencies of ESAT-6 and CFP-10 specific T cells by use of immunomagnetic enrichment [7], the fluorescence-immunospot platform [21] or longer antigen stimulation time [19]–[21]. However, the frequencies of PPD reactive T cells we detected were much higher than the frequencies of ESAT-6 and CFP-10 reactive T cells which may have made the distinction between the cytokine profiles among the patient groups substantially more discernible for the PPD specific T cells.

In conclusion, the PPD-based whole blood approach presented here may be performed from less than 1 ml of whole blood and produces results within one working day due to its short stimulation time of only 6 hours. Thus, it holds promise for being used in a routine clinical setting to rapidly allow distinction between active and successfully treated disease or latent *M. tuberculosis* infection.
